# Effect of Plastrum Testudinis Extracts on the Proliferation and Osteogenic Differentiation of rBMSCs by Regulating p38 MAPK-Related Genes

**DOI:** 10.1155/2019/6815620

**Published:** 2019-03-07

**Authors:** Qi Shang, Xiang Yu, Hui Ren, Gengyang Shen, Wenhua Zhao, Zhida Zhang, Jinjing Huang, Peiyuan Yu, De Liang, Jingjing Tang, Xiaobing Jiang

**Affiliations:** ^1^The First Clinical College of Guangzhou University of Chinese Medicine, Guangzhou, 510405, China; ^2^Lingnan Medical Research Center of Guangzhou University of Chinese Medicine, Guangzhou 510405, China; ^3^Department of Spinal Surgery, The First Affiliated Hospital of Guangzhou University of Chinese Medicine, Guangzhou 510405, China; ^4^Shanghai University of Chinese Medicine, Shanghai 200032, China

## Abstract

Extracts from plastrum testudinis (PTE) are active compounds that have been used to treat bone diseases in traditional Chinese medicine for thousands of years. In previous studies, we demonstrated their effects on glucocorticoid-induced osteoporosis both* in vivo* and* in vitro*. However, the mechanisms by which PTE regulates the osteogenic differentiation of rat bone marrow-derived mesenchymal stem cells (rBMSCs)* in vitro* remain poorly understood. In this study, rBMSCs were treated with medium (CON), PTE, osteogenic induction (OI), and a combination of PTE and OI (PTE+OI) over a 21-day period. We found that PTE significantly promoted rBMSCs osteogenic differentiation and mineralisation after 21 days of culturing. Moreover, PTE+OI further enhanced the differentiation and mineralisation process. PTE upregulated STE20, IGF1R, and p38 MAPK mRNA expression and downregulated TRAF6 mRNA expression. The extracts inhibited TRAF6 protein expression and promoted STE20, IGF1R, and phosphorylated p38 MAPK protein expression. Our results imply that PTE promotes the proliferation and osteogenic differentiation of rBMSCs by upregulating p38 MAPK, STE20, and IGF1R and downregulating TRAF6 expression, which may provide experimental evidence of the potential of PTE in the treatment of osteoporosis.

## 1. Introduction

Bone marrow-derived mesenchymal stem cells (BMSCs), which are multipotent cells with self-renewal ability, exhibit directional differentiation under appropriate stimulation [1]. As BMSCs are easily extracted and exhibit differentiation potential, cultured BMSCs are widely used* in vitro* to evaluate factors that contribute to osteogenesis [2]. Previously, we reported the beneficial effect of the extracts from plastrum testudinis (PTE) in glucocorticoid-induced osteoporosis (GIOP) in the rat spine* in vivo* [3, 4]. Furthermore, previous studies have demonstrated that PTE could promote BMSCs proliferation [5–7]. However, the underlying mechanisms by which PTE promotes the proliferation and osteogenic differentiation of rBMSCs are still poorly understood.

Mitogen-activated protein kinases (MAPKs) consist of c-Jun N-terminal kinases (JNK), p38 MAPK, and extracellular signal-regulated kinases (ERK) [8]. The MAPK cascade is a well-studied signaling pathway that regulates BMSCs differentiation during skeletal development [9–11]. Specifically, the p38 MAPK signalling pathway plays a crucial role in the inflammatory response, cell cycle, cell differentiation, and apoptosis [12]. More importantly, it has been demonstrated that the p38 MAPK pathway plays a key role in cell proliferation and osteogenic differentiation [9, 13, 14].

Sterile20 gene (STE20) functions upstream of the p38 MAPK cascade, involved in bone mineralisation [15–17]. Insulin-like growth factor 1 receptor (IGF-1R) is an upstream regulator of the p38 MAPK signalling pathway [18] and subsequently regulates cell growth, apoptosis, mineralisation, differentiation, and osteogenesis via binding of the IGF-1 ligand [19]. Tumor necrosis factor receptor-associated factor 6 (TRAF6), which also occurs upstream of the p38 MAPK pathway, has been shown to play a crucial role in regulating NF-*κ*B signalling, thus inhibiting osteoblast function [20, 21]. Given that the p38 MAPK signalling pathway and its related genes (STE20, IGF-1R, and TRAF6) play an essential role in the process of bone formation, we speculate that PTE influence the proliferation and osteogenic differentiation of rBMSCs through p38 MAPK and its related genes.

In this study, we investigated the effect of PTE on rBMSCs proliferation and osteogenic differentiation and examined the mechanism underlying p38 MAPK and its related genes in PTE-induced osteogenesis.

## 2. Results

### 2.1. Culture and Characterisation of rBMSCs

**Figure 1:**
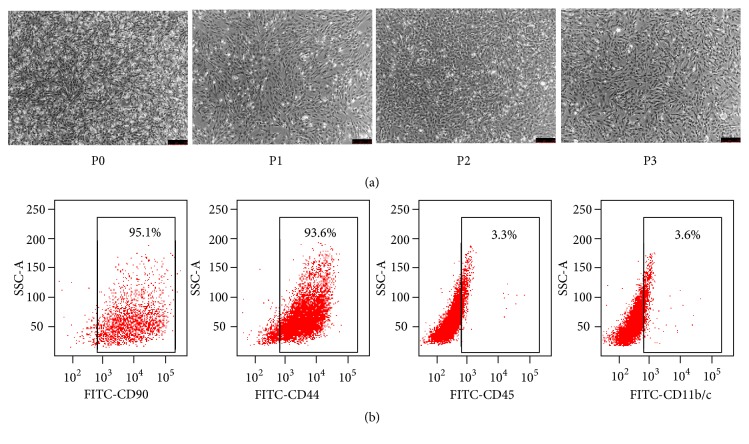
*Culture and identification of BMSCs.* (a) Changes of cell morphology of BMSCs after cultured at three passage under inverted phase contrast microscope (magnification, 50×; scale bars, 250 *μ*m). (b) Expressions of surface antigens (CD90, CD44, CD45, and CD11b/c) of BMSCs detected by flow cytometry.

rBMSCs were cultured and purified* in vitro*. After culturing for 48h, numerous adherent cells could be seen. The majority of the cells displayed long spindle-like morphology. After three passages, the size of cell colony significantly increased, and the cells presented a spindle-like form (Figure 1(a)). Flow cytometry analysis was performed to investigate the surface antigen markers of the cultured cells. The cells were CD44- and CD90-positive and negative for CD11b/c and CD45 (Figure 1(b)). Taken together, these results indicated that the isolated adherent cells were phenotypically equivalent to typical rBMSCs.

### 2.2. PTE Promoted rBMSCs Proliferation

**Figure 2:**
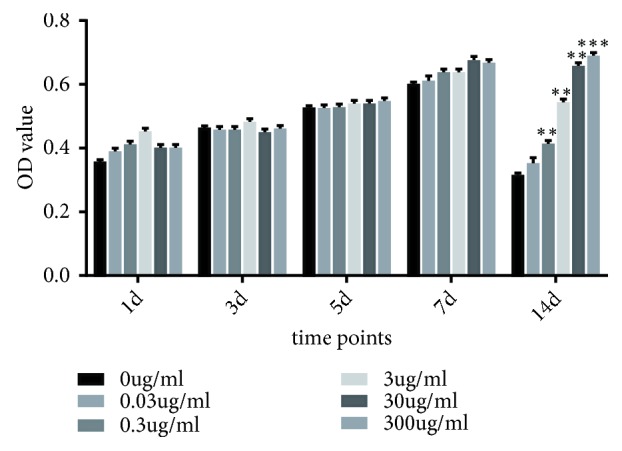
*Effect on rBMSCs proliferation of PTE was analysed at the 1* ^th^ *, 3* ^th^ *, 5* ^th^ *, 7* ^th^ *, and 14* ^th^ *day.* The values of OD 450 were measured at the specific time points. The data were expressed as mean ± SD. *∗∗P *< 0.01 and *∗∗∗P* < 0.001 versus the 0 ug/ml (control group).

rBMSCs proliferation was examined at 1, 3, 5, 7, and 14 days (Figure 2). The relative cell numbers evaluated by the optical density (OD) value at each concentration of PTE gradually increased from the first to the seventh day. Furthermore, higher OD values were observed in groups to which PTE had been applied than in the control group (0 *μ*g/mL). Additionally, rBMSCs proliferation showed a steady slight decrease at a concentration of 30 *μ*g/mL PTE on the 14^th^ day. Therefore, we selected a concentration of 30 *μ*g/mL PTE for the subsequent experiment.

### 2.3. PTE Promoted Osteogenic Differentiation of rBMSCs

**Figure 3:**
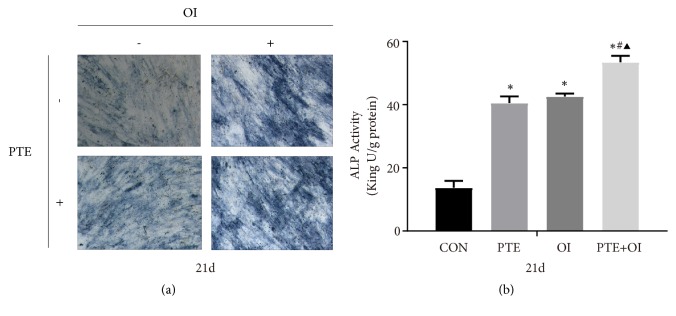
*Osteogenic differentiation assay of rBMSCs.* (a) The ALP staining assay of rBMSCs treated with PTE, osteogenic induction medium, and the combination of PTE and osteogenic induction medium at 21^th^ day (magnification, 50×; scale bar, 100 *μ*m). (b) Quantitative assay of ALP staining. The data were expressed as mean ± SD. *∗p*<0.05 versus control group, ^#^*p*<0.05 versus PTE group, and ^▲^*p*<0.05 versus OI group.

To investigate the differentiation of rBMSCs stimulated by PTE, osteogenic induction medium (OI), the combination of PTE and osteogenic induction medium (PTE+OI) and the activity of alkaline phosphatase (ALP), a marker of osteogenesis, were also examined after 21 days of osteogenic differentiation (Figure 3). After 21 days of culturing, the PTE, OI, and PTE+OI groups all showed significantly stronger ALP staining compared to the control group, with the PTE+OI group exhibiting the strongest ALP staining.

**Figure 4:**
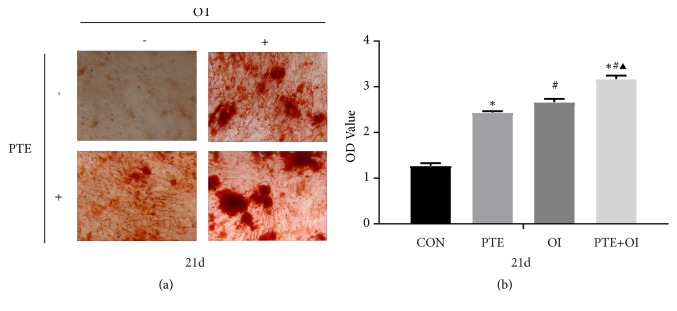
*Osteogenic differentiation assay of rBMSCs*. (a) The Alizarin red staining assay of rBMSCs treated with PTE, osteogenic induction medium, and the combination of PTE and osteogenic induction medium at the 21^th^ day (magnification, 50×; scale bar, 100 *μ*m)). (b) Quantitative assay of Alizarin red staining. The data were expressed as mean ± SD. *∗p*<0.05 versus control group, ^#^*p*<0.05 versus PTE group, and ^▲^*p*<0.05 versus OI group.

Additionally, Alizarin red staining was performed at day 21 to evaluate the osteogenic mineralisation ability of rBMSCs after applying PTE,OI, and PTE+OI (Figure 4). After 21 days, mineralized nodules had formed all three groups, with the highest matrix mineralisation in the PTE+OI group.

### 2.4. PTE Up-Regulated STE20, IGF1R, and p38 MAPK mRNA Expression, and Down-Regulated TRAF6 mRNA Expression

**Figure 5:**
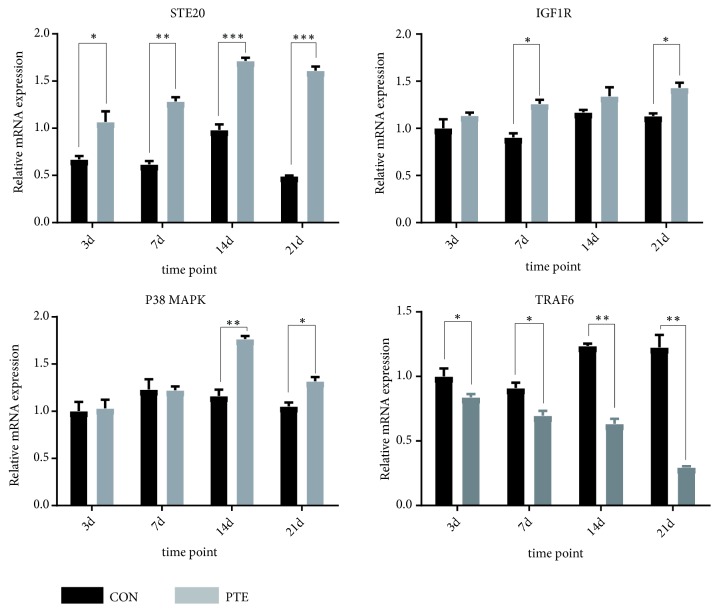
The relative mRNA expressions of STE20, TRAF6, IGF1R, and p38 MAPK were shown at the 3^th^, 7^th^, 14^th^, and 21^th^ day. The data are expressed as mean ± SD. *∗P*<0.05 versus Con group; *∗∗P*<0.01 versus Con group; *∗∗∗P*<0.001 versus Con group.

**Figure 6:**
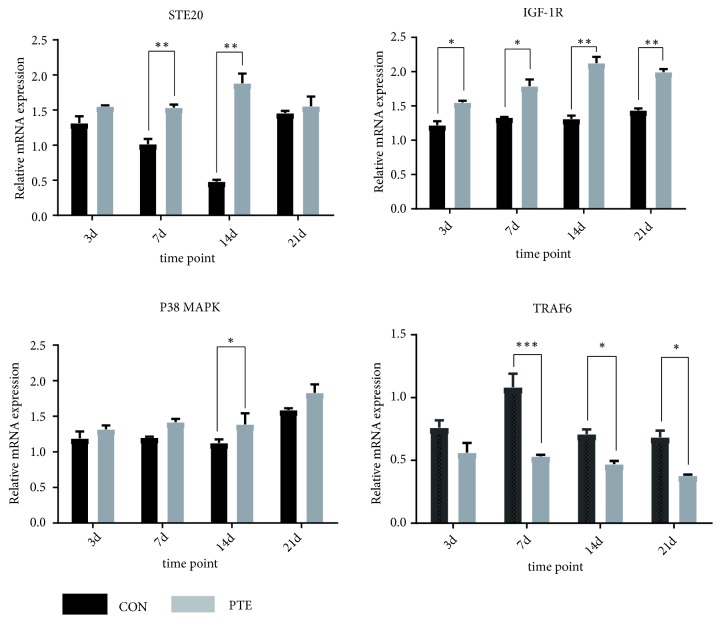
The relative mRNA expressions of STE20, TRAF6, IGF1R, and p38 MAPK were shown at the 3^th^, 7^th^, 14^th^, and 21^th^ day. The data are expressed as mean ± SD. *∗P*<0.05 versus OI group; *∗∗P*<0.01 versus OI group; *∗∗∗P*<0.001 versus OI group.

The relative mRNA expression of STE20 was significantly higher in the PTE group than in the control group at four time points. The relative mRNA expressions of IGF1R and p38 MAPK were upregulated in PTE group compared to those in the control group on days 3, 7, 14, and 21, whereas the relative mRNA expression of TRAF6 was downregulated at all four time points in the PTE group compared to the control group. Additionally, after osteogenic induction, the relative mRNA expressions of STE20, IGF1R, and p38 MAPK were upregulated at all four time points in the OI+PTE group. In contrast, the relative mRNA expression of TRAF6 was generally downregulated in the OI+PTE group (Figures 5 and 6).

### 2.5. PTE Upregulated STE20, IGF1R, and p-P38 MAPK Protein Expression and Downregulated TRAF6 Protein Expression

**Figure 7:**
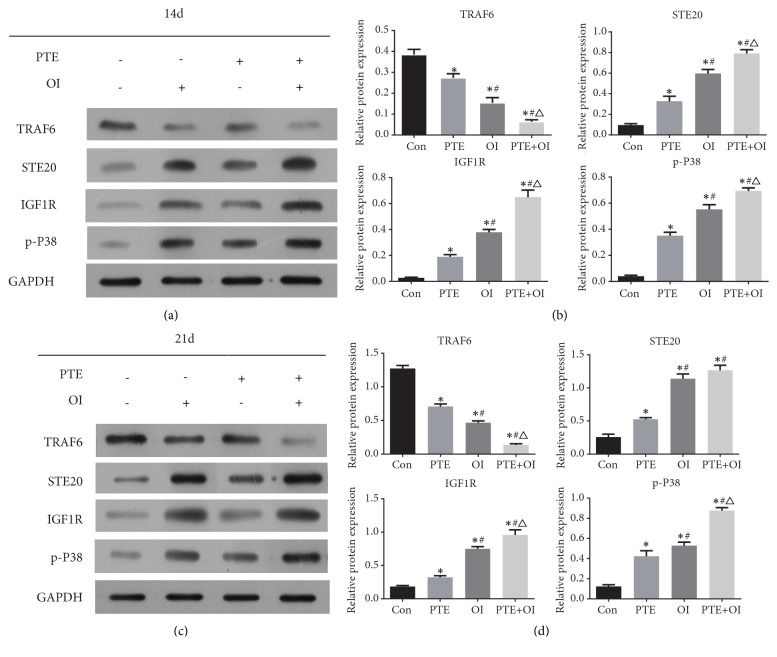
Protein expression levels of TRAF6, STE20, IGF1R, and p-p38 MAPK were analysed by Western Blot. The data are expressed as mean ± SD. ^*∗*^*P*<0.05 versus control group, ^#^*P*<0.05 versus PTE group, and ^△^*P*<0.05 versus OI group.

After osteogenic induction at 14 and 21 days, the protein expressions of STE20, IGF1R, and p-P38 in all three groups, PTE, OI, and OI+PTE, were significantly upregulated compared to the control group. Conversely, TRAF6 protein expression was downregulated compared to the control group during rBMSCs osteogenic differentiation (Figure 7).

## 3. Discussion

The skeleton is a highly dynamic tissue whose structure relies on the balance between bone deposition and resorption [22]. Currently, most of the drugs available for osteoporosis are aimed at inhibiting osteoclastic bone resorption, and only a few drugs promote osteoblastic bone formation [23]. Therefore, it is becoming increasingly important to identify the factors that regulate bone formation and to develop new drugs that promote this process. PTE extracts are active compounds of plastrum testudinis, which have been used to treat bone diseases in traditional Chinese medicine for thousands of years.

In our study, we explored the preliminary mechanism underlying the promotion of the osteogenic differentiation of rBMSCs by PTE. As the culture medium plays an important role in cell differentiation, we examined the PTE-promoted osteogenesis of rBMSCs in osteogenic medium. rBMSCs cultured in normal medium with PTE and in OI with PTE showed increased ALP activity and calcium deposition at 21 days, indicating that PTE promoted the osteogenic differentiation of rBMSCs and that PTE combined with OI had a synergistic effect on differentiation and mineralisation, which was consistent with our previous study [7]. Furthermore, to investigate the underlying mechanism, we examined the mRNA and protein expression of p38 MAPK and its related genes (STE20, IGF1R, and TRAF6) at different time point during rBMSCs osteoblast differentiation.

Previous studies have reported the crucial role of p38 MAPK in bone formation* in vivo* [24]. Attenuation of p38 MAPK phosphorylation* in vitro* inhibited osteoblast migration and differentiation [25–27]. In our study, we used quantitative polymerase chain reaction (QT-PCR) assays to show that PTE upregulated p38 MAPK expression and Western blot analysis to show that it upregulated p38 MAPK phosphorylation, indicating that PTE promotes osteoblast differentiation via p38 MAPK. Additionally, we examined several genes related p38 MAPK for further confirmation of this process.

**Figure 8:**
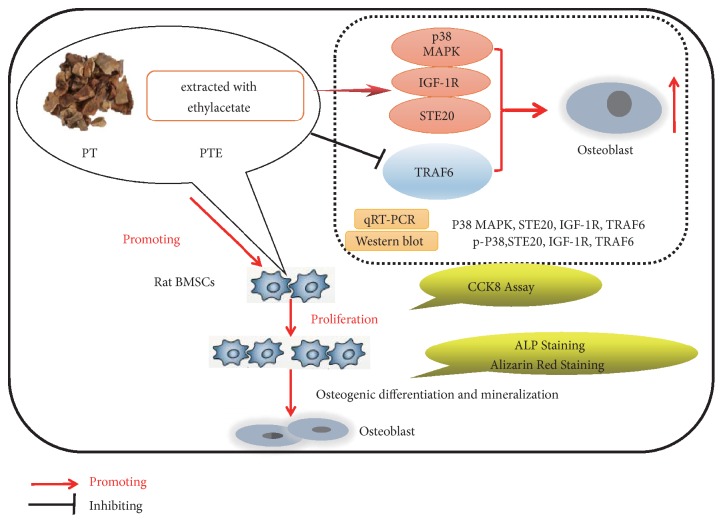
Schematic of PTE on proliferation and osteogenic differentiation of rBMSCs by regulating p38 MAPK-related genes based on our findings.

MAPK signalling pathways alter the expression, localisation, binding ability, and stability of transcriptional regulators [12]. The various MAPK pathways share a common family of upstream mediators such as STE20, IGF1R, and TRAF6. The STE20 kinases function as MAP4Ks, triggering activation of the MAPK cascade, which can activate p38 MAPK [28]. Furthermore, it has been reported that STE20 is a regulator of bone mineralisation [17]. IGF-1, whose receptor is IGF1R, was shown to be essential for matrix biosynthesis to sustain mineralisation and to play an important anabolic role in stimulating bone formation and maintaining bone mass [29]. A decrease in IGF-1R expression caused decreases in the amount of phosphorylated p38 MAPK and inhibited the p38 MAPK signalling pathway [18, 30]. Furthermore, mutation of IGF1R correlated with a striking reduction in bone volume and trabecular number and an increase in trabecular spacing [29]. TRAF6 was shown to activate NF-*κ*B signalling, which suppressed osteoblast function and promoted osteoclast formation [20]. Additionally, a decrease in TARF6 resulted in an increase in the number of osteoblasts [31]. In our study, we observed increased expression of IGF1R and STE20 and decreased expression of TRAF6, implying that PTE promotes the proliferation and osteogenic differentiation of rBMSCs by regulating p38 MAPK-related genes and proteins such as STE20, IGF1R, and TRAF6 (Figure 8).

## 4. Materials and Methods

### 4.1. Cell Isolation and Culture

rBMSCs were obtained from a 2-week-old Sprague-Dawley (SD) male rat. Bone marrow was flushed out of the femur and tibia with SD rBMSC basal medium (Cyagen, Guangzhou, China) supplemented with 10% (v/v) Sprague-Dawley (SD) rBMSC cell-qualified Fetal Bovine Serum, 1% (v/v) penicillin-streptomycin (Cyagen, Guangzhou, China), and 1% (v/v) glutamine in standard sterile condition. Cells were plated and then incubated in a humidified atmosphere of 5% CO_2_ at 37°C. They were passaged every 3 days using 0.25% (w/v) trypsin-EDTA solution (Cyagen, Guangzhou, China). After the third or fourth passage, the cells were used in our experiments [32].

The rBMSCs were cultured in an osteogenic induction medium (Cyagen, Guangzhou, China), composed of 175 mL SD rBMSC Basal Medium, 20 ml cell-qualified fetal bovine serum, 20 uL dexamethasone, 400 uL ascorbate, 2 mL glutamine, 2 mL penicillin-streptomycin, and 2 mL *β*-glycerophosphate, at an initial density of 2×10^4^ cells/cm^2^ (with 1 mL osteogenic induction medium per well) in 12 well plates to induce osteogenic differentiation. The medium was changed every 3 days during osteogenesis. The cells were observed under an inverted phase contrast microscope (Leica, Solms, Germany)

### 4.2. Cell Surface Marker Characterised by Flow Cytometry

The rBMSCs were characterised using flow cytometry. Third-passage cells adherent to 80% confluence were selected, digested with 0.25% (w/v) trypsin-EDTA solution (Cyagen, Guangzhou, China), centrifuged at 250rcf for 5 min at 4°C, and suspended at 3× 10^6^/ml using PBS containing 0.1% bovine serum albumin (BSA). Next, 100ul of the cell suspension was placed into 1.5 mL Eppendorf (each with 100ul). Monoclonal antibodies (CD90, CD44, CD45, and CD11b/c) (Cyagen, Guangzhou, China) from the rBMSCs were added to each Eppendorf, and mouse IgG1 was used as the isotype control antibody. After incubation in the dark at 4°C for 30 min, FITC-conjugated goat anti-mouse IgG (Cyagen, Guangzhou, China) was labelled with CD90, CD44, CD45, and CD11b/c; this was followed by incubation in the dark at 4°C for 30 min. Then, flow cytometry analysis was performed using a FACS Canto TM II Flow Cytometer (BD Bioscience, San Jose, CA, USA).

### 4.3. Preparation of Plastrum Testudinis Extracted with Ethylacetate

PTE extracts were obtained according to a previously established method [5]. The concentrations of PTE used in the study were 0 (as control group), 0.03, 0.3, 3, 30, and 300 *μ*g/mL.

### 4.4. Cell Counting Kit 8 (CCK-8) Assay

rBMSCs with a density of 5, 000 cells/well were seeded in 96 well plates and cultured in growth medium. The proliferation of rBMSCs was assessed with the cell counting kit-8 (Tokyo, Japan) by using an OD setting of 450 nm in the microplate reader (Varioskan Flash; Thermo Fisher Scientific, Waltham, MA, USA) following the manufacturer's protocol [33].

### 4.5. Osteogenic Differentiation Protocol

The optimal concentration of PTE for inducing osteogenic differentiation was selected based on the results of the CCK8 assay. To induce osteogenic differentiation, rBMSCs were cultured in growth medium (Cyagen, Guangzhou, China) in 12-well cell culture plates (Corning, Shanghai, China), at a density of 2 ×10^4^/cm^2^ and incubated for 48h at 37°C under 5% CO_2_. Cells were divided into the following four groups: Group A: control group, which received rBMSC basal medium (Cyagen, Guangzhou, China); Group B: PTE group, which received rBMSC basal medium with PTE; Group C: OI group, which received osteogenic induction medium; and Group D: PTE+OI group, which received osteogenic induction medium with PTE.

### 4.6. Alkaline Phosphatase Assay

rBMSCs were harvested at 21 days and re-suspended in PBS (pH 7.4), followed by homogenisation with ultrasonication. After centrifugation, ALP activity in the supernatants was assessed using ALP detection kit (Nanjing Jiancheng Bioengineering Institute, Nanjing, China), reading the absorbance value on the microplate reader at 520 nm. A BCA protein assay kit (Keygen, Nanjing, China) was used to assess total protein content, which was used for ALP activity normalisation.

### 4.7. Alizarin Red Staining

After culturing in osteogenic medium for 21 days, PBS (pH 7.4) was used to wash the rBMSCs. Then, 4% paraformaldehyde was used to fix the rBMSCs at room temperature for 30 min, followed by rinsing in distilled water and then incubation for 10 min at room temperature with 40 mM Alizarin red staining (pH 4.2) (Sigma-Aldrich, Darmstadt, Germany) incubated. Observation was performed under an inverted microscope (Olympus, Tokyo, Japan). Subsequently, quantitative analysis of mineralised nodule areas indicated by the Alizarin red staining was performed using Image J software (National Institute of Health, Maryland, USA).

### 4.8. Quantitative Real-Time PCR (qRT-PCR)

rBMSCs were harvested for RNA extraction. Total RNA was isolated from the rBMSCs using Trizol reagents (Invitrogen, Carlsbad, CA, USA). Polymerase chain reaction (PCR) was performed using the SYBR Green PCR kit (Invitrogen, Carlsbad, California, USA) from Applied Biosystems. GAPDH was used for gene expression normalisation. The primer sequences in the experiment are described in Table 1. The 2^-△△Ct^ method was used to calculate the relative gene expression. All reactions were performed in triplicate.

### 4.9. Western-Blot Analysis

Protein extracts from cells were prepared in RIPA lysis buffer supplemented with a proteasome inhibitor (Beyotime, Haimen, China). Total proteins were separated by using 10% SDS-PAGE and then transferred to a PVDF membrane (Millipore, Shanghai, China). After blocking in 5% BSA for 1 h at room temperature, the membranes were incubated overnight at 4°C with antibodies specific to GAPDH (RC-5G5; 1:10000; KangChen Bio-tech, Shanghai, China), TRAF6 (ab33915; 1:8000; Abcam, Cambridge, UK), p-p38 MAPK (#4511; 1:1000; CST, Boston, USA), STE20 (ab155198; 1:3000; Abcam, Cambridge, UK), and IGF1R (ab79176; 1:2000; Abcam, Cambridge, UK). HRP goat anti-rabbit IgG (BA1054; 1:20000, Boster Biologic Technology, Wuhan, China) was used as a secondary antibody for 2 h at room temperature. The signal intensity was quantified using Image J software (National Institute of Health, Bethesda, Maryland, USA).

### 4.10. Statistical Analysis

The data were analysed with SPSS 19.0 software (SPSS Inc., Chicago, IL, USA). All results were expressed as means ± standard deviations (SDs). Comparisons between two groups were performed by Student's t-test, whereas comparisons among the groups were performed using one-way analysis of variance (ANOVA) with Turkey's post hoc test. Two-sided* P*-values < 0.05 were considered statistically significant.

## 5. Conclusion

Our results indicate that PTE could promote the proliferation and osteogenic differentiation of rBMSCs. Furthermore, the combination of PTE and osteogenic induction medium further enhanced osteogenic differentiation. Additionally, this study indicates that p38 MAPK and its related genes (STE20, IGF1R, and TRAF6) are involved in the mechanism of rBMSCs' osteogenic differentiation, which provides experimental evidence of the potential application of PTE in the treatment of osteoporosis. However, further research is necessary to elucidate the details of the upstream and downstream process of the p38 MAPK signalling pathway. Furthermore, the application of advanced techniques, such as gene overexpression and knock-out studies, should be examined in future studies.

## Figures and Tables

**Table 1:** Quantitative PCR primer sequences.

Gene	Forward (5′-3′)	Reverse (5′-3′)
TRAF6	TGACAATGAAATACTGCTGGAA	CACAGCCTTTATTTGGACACTT
p38 MAPK	GATATTTGGTCCGTGGGC	TGGCTTGGCATCCTGTTA
STE20	AACCAAGGACAGTGGCTCG	CTGGGCTTTCTGCTCTTCC
IGF1R	ACATCCGCAACGACTATCAG	TTGTAGAAGAGTTTCCAGCCAC
GAPDH	CCTCGTCTCATAGACAAGATGGT	GGGTAGAGTCATACTGGAACATG

## Data Availability

The data used to support the findings of our study are available from the correspondences upon request.

## Abbreviations

PTE: Plastrum testudinis extracts
rBMSCs: Rat bone marrow-derived mesenchymal stem cells
OI: Osteogenic induction
MAPK: Mitogen-activated protein kinase
p-p38: Phosphorylated p38
NF-*κ*B: Nuclear factor-*κ*B
STE20: Sterile20
IGF1R: Insulin-like growth factor 1 receptor
TRAF6: Tumor necrosis factor receptor-associated factor 6
ALP: Alkaline phosphatase
GAPDH: Glyceraldehyde 3-phosphate dehydrogenase
ARS: Alizarin red staining.
